# Effect of charge and telechelic polymer on the structural ordering and stability of neutral lamellar phases: insights from small-angle neutron scattering on TX_100_/TX_35_ systems

**DOI:** 10.1039/d5ra05970d

**Published:** 2025-11-20

**Authors:** Z. Basbassi, M. Khatouri, L. Talha, R. Ahfir, A. Arbia, R. Elhajjam, S. El Khaoui, M. Filali

**Affiliations:** a Laboratory of Advanced Materials and Applications (LM2A), Faculty of Sciences Dhar El Mahraz, Sidi Mohamed Ben Abdellah University Fez-Sais Morocco mohammed.khatouri@usmba.ac.ma

## Abstract

Small-angle neutron scattering (SANS) experiments were conducted in this study to investigate the structure and stability of a neutral lamellar phase. This phase consists of Triton X_100_ (TX_100_) as the primary surfactant, Triton X_35_ (TX_35_) as the co-surfactant, and water. To prepare samples within the lamellar phase region, the mass ratio was fixed at *Ω* = TX_35_/TX_100_ = 1.26. All scattered intensities, *I*(*q*), were fitted using the Nallet model, which provides insight into the spatial arrangement of membranes in the system. We first focused on the effect of the volume fraction *ϕ* on the structure and stability of the lamellar phase. Subsequently, a cationic surfactant, cetylpyridinium chloride (CpCl), was added at a fixed concentration of *Γ* = 1% to samples with different volume fractions in order to assess the influence of introducing charges on the lamellar structure. Finally, a telechelic polymer, polyethylene oxide modified with hydrophobic alkyl (dodecyl) groups at both chain ends (denoted PEO-2m), was incorporated at a fixed concentration of *Ψ* = 10% into samples of various volume fractions. The results reveal that neither the variation of the volume fraction nor the addition of charges or PEO-2m alters the phase type: all samples remain in the lamellar phase. However, the internal structure is strongly affected. At low volume fractions (*ϕ* = 5% and *ϕ* = 10%), the membranes are disordered and lack a well-defined lamellar arrangement, whereas at higher volume fractions (*ϕ* = 20% and *ϕ* = 30%), they adopt an ordered state typical of a well-organised lamellar structure. The introduction of charges or PEO-2m significantly enhances the structural order: a well-defined lamellar phase is obtained even at low volume fractions (*ϕ* = 5% and *ϕ* = 10%) in the presence of charges, and at *ϕ* = 10% when PEO-2m is added.

## Abstract 2

Small-angle neutron scattering (SANS) experiments were conducted in this study to investigate the structure and stability of a neutral lamellar phase.

## Graphical abstract

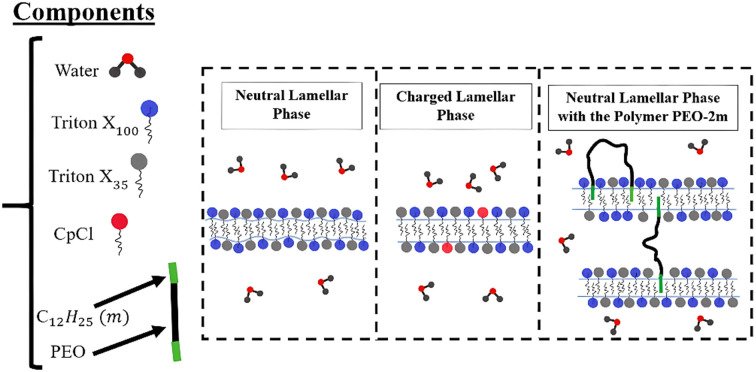

## 1. Introduction

A wide variety of mesophases are formed when amphiphilic molecules self-assemble in solution due to the balance between hydrophobic and hydrophilic interactions: micelles, vesicles, rods, sponge phases, and lamellar phases.^1–11^ The properties of these self-assembled structures are highly sensitive, with their morphology and stability being influenced by factors such as pressure, temperature, constituent ratios, and interaction strength. Owing to their structural simplicity, lamellar phases have attracted particular attention because of their crucial role in biological systems.^12–16^ They exhibit a simple yet adaptable organisation, consisting of alternating bilayers of surfactant molecules separated by thin solvent layers and periodically stacked, forming a one-dimensional arrangement. This configuration, which closely resembles the structure of biological membranes, plays an essential role in numerous applications, including drug delivery. For this reason, active molecules are often incorporated, and the presence of a third component typically modifies the phase behaviour of the lyotropic system.^17–20^ Lamellar phases are stabilised by various repulsive forces acting between adjacent membranes, among which a significant contribution arises from thermally induced membrane undulations. According to Helfrich,^21,22^ the spatial confinement of fluctuating bilayers between neighbouring layers generates a steric repulsion of entropic origin. This fluctuation-induced repulsion is highly dependent on the bending rigidity of the membranes, described by the bending modulus *κ*, which governs the amplitude of thermal undulations and, consequently, the effective interlayer interactions.^23–25^

In neutral lamellar systems, the stability is generally attributed to the Helfrich repulsion, which arises from thermal fluctuations of flexible bilayers. This mechanism, well established in the literature,^26^ provides a theoretical framework for understanding the observed lamellar stability.

The incorporation of charged species, either *via* ionic surfactants or added salts, introduces new electrostatic interactions that can significantly modify the properties of the lamellar phase. Charged additives can alter the bilayer spacing, induce phase transitions, or even lead to the formation of new mesophases. These effects arise from variations in osmotic pressure, membrane bending rigidity, and electrostatic repulsion between layers. In contrast to neutral systems, charged lamellar phases often display more complex behaviour, such as limited swelling or undulation-induced instabilities.^27–32^

Several studies have highlighted the impact of electrostatic interactions in lamellar systems. For instance, Oberdisse *et al.*^27^ and Porte *et al.*,^11^ investigating a non-ionic ternary surfactant system doped with small amounts of an ionic surfactant, demonstrated that the presence of charged surfactant ions can markedly modify the phase and structural behaviour compared with neutral systems. Neutron scattering data revealed that, at very low concentrations, small vesicular phases emerge, whereas at moderate concentrations, an elastic onion-like phase develops. Polymers constitute another particularly effective class of additives for tuning the structure and inter-bilayer interactions of lamellar phases. Numerous studies have investigated the influence of different types of polymers. For example, neutral water-soluble polymers such as poly(ethylene glycol) (PEG)^33–35^ or poly(vinylpyrrolidone) (PVP) can induce either steric repulsion or depletion attraction, depending on their concentration and molecular weight.^36,37^ Comparative studies have shown that polymer–bilayer interactions may lead to both enhanced rigidity and lamellar disorder, depending on the system parameters. Ficheux *et al.*^38^ examined the influence of two neutral water-soluble polymers, polyacrylamide (PAM) and PEG, on the stability and structure of a charged lamellar phase formed by sodium di-2-ethylhexylsulfosuccinate (AOT) and water, using small-angle neutron scattering (SANS) and X-ray scattering. PEG, an adsorbing polymer, strongly modified the thermodynamic and structural properties of the AOT lamellar phase, inducing significant changes in Bragg peak intensity and broadening, suggesting a pronounced impact on bilayer interactions and compressibility (reduction of the compression modulus *B*). Conversely, PAM, a non-adsorbing polymer, behaved almost inertly, exerting minimal influence on lamellar structure and interactions, with only weak effects on both the compression modulus *B* and curvature modulus *K*. An especially intriguing case involves the incorporation of telechelic polymers bearing hydrophobic end groups. These polymers anchor into the surfactant bilayers *via* their hydrophobic terminal groups (“stickers”), while the hydrophilic mid-chain segments either bridge adjacent layers or remain solvated in the aqueous interlamellar space.^39,40^ Compared with simple linear polymers, telechelic polymers induce unique interactions owing to their ability to simultaneously anchor into multiple bilayers. Their incorporation can generate bridging interactions and entropic constraints, acting as molecular connectors that markedly influence the lamellar architecture. These effects can modify membrane spacing, swelling behaviour, long-range order, bilayer elasticity, and even phase stability. The nature of the surfactant matrix also plays a crucial role in determining these interactions. Non-ionic surfactants, such as Triton X_100_, are particularly attractive for such systems due to their chemical stability, low toxicity, and insensitivity to ionic strength and pH.^41–45^ Triton X_100_, consisting of an aromatic hydrophobic tail and a polyethylene oxide (PEO) hydrophilic headgroup, readily forms ordered lamellar structures over a wide concentration range in aqueous solution without losing structural order. This makes it an ideal platform for incorporating additives such as polymers or charged surfactants to tailor its physical properties.^46^ Unlike ionic surfactants, where long-range electrostatic interactions dominate, the lamellar phases of non-ionic surfactants like Triton X_100_ are primarily governed by steric repulsion, hydration forces, and thermal fluctuations, rather than inter-bilayer electrostatics. These interactions control interlamellar spacing, elasticity, and phase stability, and they can be finely modulated through the addition of co-solutes or external stimuli.

**Fig. 1:**
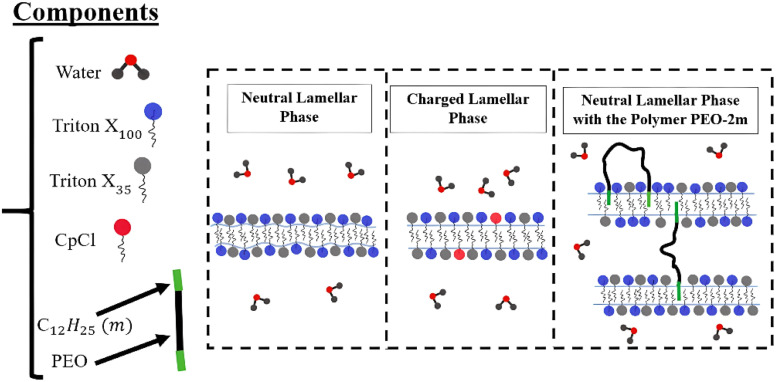
Schematic illustration summarising the key steps and main findings of the study.

Several studies have investigated the properties of lamellar phases. In this context, Chih-Ying Liu and Hsin-Lung Chen^47^ established that dendritic polymers can induce undulated lamellar structures by modulating interfacial curvature and balancing elastic energy. In contrast, our work demonstrates that PEO-2m, a linear polymer, can stabilise a well-ordered lamellar phase with a clearly defined periodicity. This difference highlights the crucial role of polymer architecture and binding mode in determining lamellar morphology and structural stability. Similarly, Agzenai Y. *et al.*^48^ reported that introducing the monomer diallyl dimethylammonium chloride into an AOT/water system perturbs the lamellar phase by localising in the water layers, modifying the hydration shell, and inducing electrostatic shielding of the charged bilayers. This process reduces bilayer rigidity and alters undulation interactions, leading to a partial transition towards the L_*α*_ sponge phase. Furthermore, the study on double-end-anchored PEG–lipids^49^ demonstrated the reversible control of lamellar spacing through polymer bridging and tethering, effectively locking membrane distances in charged hydrogels. Consistent with these findings, our results show that the bridging effect of PEO-2m contributes significantly to the stabilisation of the neutral lamellar phase. Overall, to the best of our knowledge, this is the first SANS-based investigation demonstrating that a neutral surfactant/water system can form a stable, ordered lamellar structure purely through the incorporation of a linear telechelic polymer, without relying on electrostatic interactions. This finding provides new insight into the role of polymer topology in modulating lamellar stability. Furthermore, by identifying and analyzing each parameter of Nallet *et al.* model, providing quantitative characterization of the lamellar structure. In this paper, we employ small-angle neutron scattering (SANS) experiments to investigate the effect of the volume fraction *ϕ* on the structure and stability of a neutral lamellar phase composed of water and the surfactants TX_100_ and TX_35_ (see Fig. 1). We then examine the influence of introducing a small amount of charge *Γ*, by adding the cationic surfactant CpCl, on the structure and stability of the lamellar phase at different volume fractions *ϕ*. Finally, we explore the effect of adding a polymer, poly(ethylene oxide) modified with hydrophobic alkyl (dodecyl) groups at both ends, referred to as PEO-2m, on the structure of the neutral lamellar phase.

## 2. Theoretical background

The stability of lamellar phases is influenced by a number of factors, including interlayer interactions. These include entropic contributions (such as thermal fluctuations described by the Helfrich interaction), steric interactions, repulsive electrostatic forces in the case of charged membranes, and attractive van der Waals forces. The balance between these different interactions determines the interlamellar distance, the structural order and the overall stability of the system. Accordingly, for a neutral lamellar phase, the lamellar stacking can be stabilized by the thermal undulations of the bilayers, which counteract the van der Waals attraction, thereby preventing phase separation the resulting force due the undulations of the bilayers of such a swollen stacking were described by Helfrich,^21,22,50,51^ this entropic interaction is given by:1
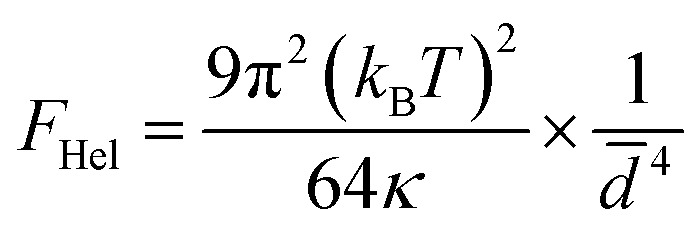
where the *d̄* = *d* − *δ* is the average thickness of the water layer between two adjacent bilayers, *k*_B_ is Boltzmann's constant, *T* is temperature, and *κ* is the membrane bending modulus. The absence of the Bragg peaks in the most diluted samples can thus understood as a consequence of these large fluctuations, which disrupt the regular lamellar stacking and reduce the contrast needed for well-defined scattering peaks.

From the experimental point of view, another relevant detail on the elastic properties of the membranes can be obtained from the small angel scattering, by estimate to the Caille parameter *η*, which reflects the bilayer fluctuations in lamellar phases. From the shape and broadening of the Bragg peaks. Indeed, the Caille parameter is related to the two the compressional modulus *B*, and *κ* the membrane bending modulus through:^52^2
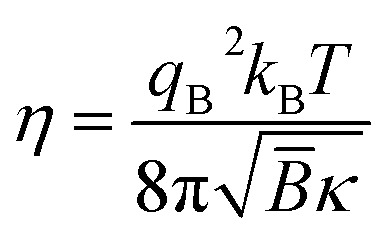
where *q*_B_ is the wave vector of the first-ordered Bragg peak, moreover when the interlamellar forces are dominant by the Helfrich interaction given by eqn (1), the Caille parameter can expressed as follow with *m* = 4/(3π^2^):^52^3
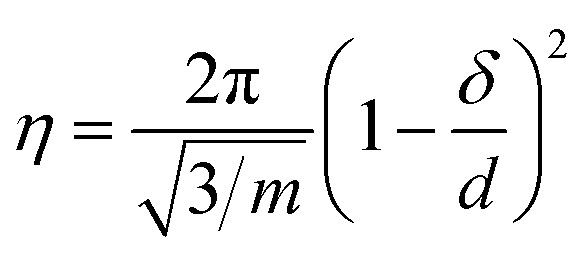


In the case of weakly screened electrostatic interactions between adjacent bilayers with bending modulus *κ*, moreover a system with solvent free from added salt the theoretically a layer compression modulus given by:^53,54^4
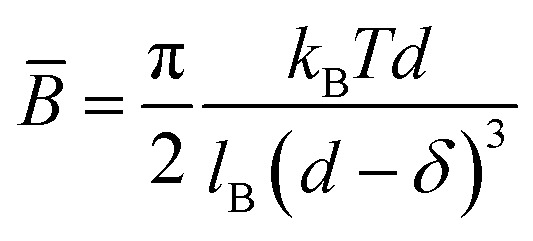
where *l*_B_ is the Bjerrum length of the solvent, for water about 0.7 nm.^53,54^

To theoretically describe the spectra obtained from small-angle neutron scattering of a lamellar phase, Nallet proposed a developed model.^50,55,56^ It considers the scattered intensity *I*(*q*) as the sum of two contributions: a diffuse scattering arising from surfactant concentration fluctuations within the membranes, and a structured scattering related to the regular stacking of the lamellar layers. This scattered intensity is given by:5
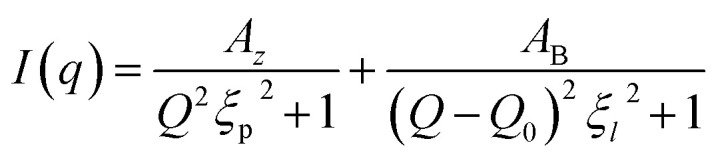
where *A*_*z*_ and *A*_B_ are amplitude factors for small angle and Bragg scattering, *ξ*_p_ is the correlation length of concentration fluctuations, *Q*_0_ is the Bragg peak position and *ξ*_*l*_ is the interlamellar correlation length. This model is applicable to dilute lamellar phases, thus by discussing the fitted parameter from the intensity profile gives us information about the structure.

## 3. Experimental section

### 3.1. Materials

The neutral surfactant Triton X_100_, cosurfactant Triton X_35_ and polyethylene oxide (PEO) was purchased from Fluka as received. As an additive, the ionic surfactant chosen is Cetylpyridinium Chloride (CpCl) purchased also from Fulka and purified along several recrystallisation in water and acetone. Furthermore, the hydrophobically modified poly(ethylene oxide) was used in the system, which contains an isocyanate group between the alkyl chain C_12_H_25_ and the ethylene oxide chain. The samples are defined by the volume fraction *ϕ*, the cosurfactant to surfactant mass ratio *Ω*, the relative amount of charge *Γ*, and the PEO-2m polymer concentration, denoted as *Ψ*. Details regarding the components are provided in Table 1.

**Table 1:** Molar mass and density of the components of the samples

Components	Molar mass (dalton)	Density (g cm^−3^)
H_2_O	18	1
[H_3_C(CH_2_)_15_]C_5_H_5_N^+^Cl^−^ (CpCl)	339.5	1.656
(H_3_C)_3_C(CH_2_)C(CH_3_)_2_C_6_H_6_(OCH_2_CH_2_)_9.5_OH (TX_100_)	624	1.07
(H_3_C)_3_C(CH_2_)C(CH_3_)_2_C_6_H_6_(OCH_2_CH_2_)_3_OH (TX_35_)	338	1.02
[CH_3_(CH_2_)_11_]–NHCO(OCH_2_CH_2_)_227_O(CO)NH–[CH_3_(CH_2_)_11_] (PEO-2m)	10 400	1.2

### 3.2. Samples preparation

Our experimental system consists to study the lamellar phase composed by a ternary mixture of non-ionic commercial surfactant Triton X_100_ (TX_100_), Triton X_35_ (TX_35_) and water. Based on stability of the lamellar phase region in the phase diagram the weight ratio of *Ω* = TX_35_/TX_100_ = 1.26 was kept constant in all samples. In addition, the samples of the neutral lamellar phase were prepared by mixing the amount of TX_100_, TX_35_, and water. This leads to a homogenous samples exhibit high viscosity and a progressive decrease in birefringence owing to the increasing in the TX_35_/TX_100_ ratio. The fluid membrane then doped by two different components at first by a cationic surfactant, cetylpyridinium chloride (CpCl), by adding a fixed amount *Γ* = 1% of this surfactant into the samples in order to investigate the effect of charge by increasing the electrostatic interaction between the membrane. Then, by the incorporation a fixed concentration *Ψ* = 10% of water-soluble neutral polymer (PEO-2m). The samples were prepared and left for several weeks at 20 °C to reach the stabilization of samples. The system was characterized by using small angle neutron scattering technique.

Or each sample, the relevant definitions outlined below are used:6
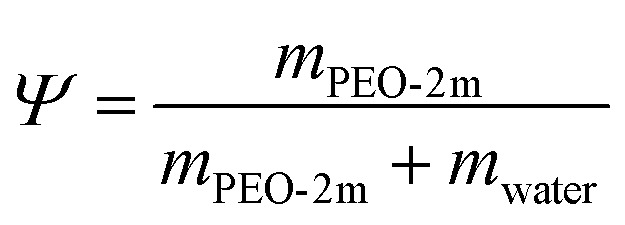
7
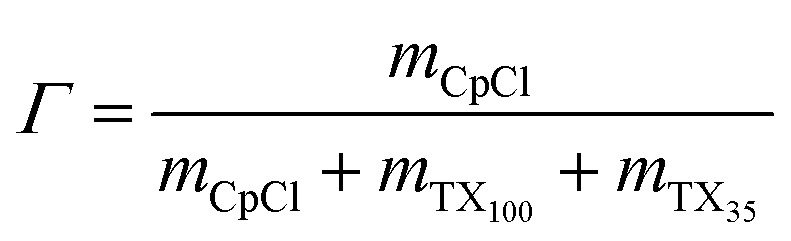
8
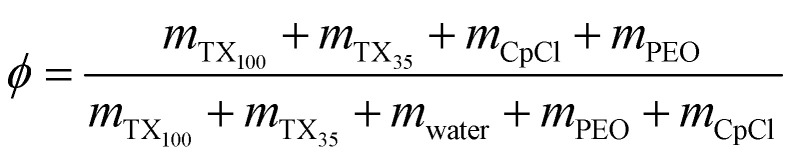
9
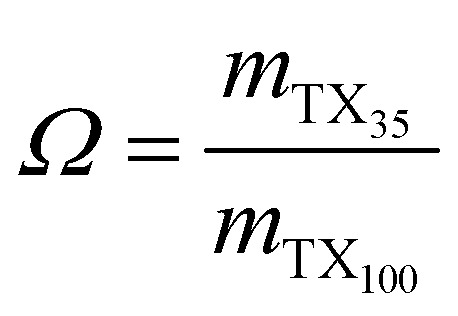


### 3.3. Small angle neutron scattering (SANS)

The small angle neutron scattering experiments were conducted at Laboratoire Léon-Brillouin in Saclay with spectrometer PAXY, (the wave vector *q* varied from 0.002 Å^−1^ to 0.4 Å^−1^), thus a beam wavelength chosen by a mechanical selector *λ* = 8 Å and (Δ*λ*/*λ* is about 3%). In this experiments we used a neutron beam width of 1 × 10^−3^ Å^−1^, 2.8 × 10^−3^ Å^−1^ and 5 × 10^−3^ Å^−1^,with decreasing sample-to- detector distance, samples were carried out in 1 to 2 mm optical path length quartz cells (Hellma). As a supplementary analytical method, polarizing microscopy observations were performed to validate whether the samples have a typical behavior of lamellar phases.

## 4. Results and discussions

In this study, we use small-angle neutron scattering (SANS) experiments on samples composed of TX_35_, TX_100_, and water. The mass ratio is fixed at *Ω* = TX_35_/TX_100_ = 1.26 for all samples in order to obtain systems within the lamellar phase region. We investigate the effect of the volume fraction *ϕ*, the addition of a certain amount of charge, and the incorporation of a polymer on the structure of the lamellar phase. To confirm that the scattered intensities *I*(*q*) correspond to a lamellar phase structure, and to better understand the influence of these three parameters on this structure, all scattering intensities are fitted using Nallet's equation (eqn (5)). In general, to validate that a sample exhibits a lamellar phase, the scattered intensity *I*(*q*) must be well described by Nallet's equation. Such intensity typically exhibits a Bragg peak when the system is ordered and decays monotonically with the scattering vector *q* when no long-range order is present.

### 4.1. Phase behavior

We focus on a particular series of samples where the volume fraction of water *ϕ* ranges between 5% and 30%. By increasing the co-surfactant/surfactant (*Ω*) ratio there's sequences of distinct phases appear. At low value of *Ω* (*Ω* < 0.3), the system forms the micellar phase, it's a transparent phase characterized by the absence of the birefringence. Upon further increasing *Ω* a biphasic region appears involve the micellar phase and the lamellar phase, beyond this, in the range of *Ω* = 0.6 and *Ω* = 1.5 a well-defined region of the lamellar phase is observed, the samples in this range exhibit increased viscosity, and a strong birefringence between two polarizers.

### 4.2. Effect of volume fraction on the structural properties of a neutral lamellar phase

**Fig. 2:**
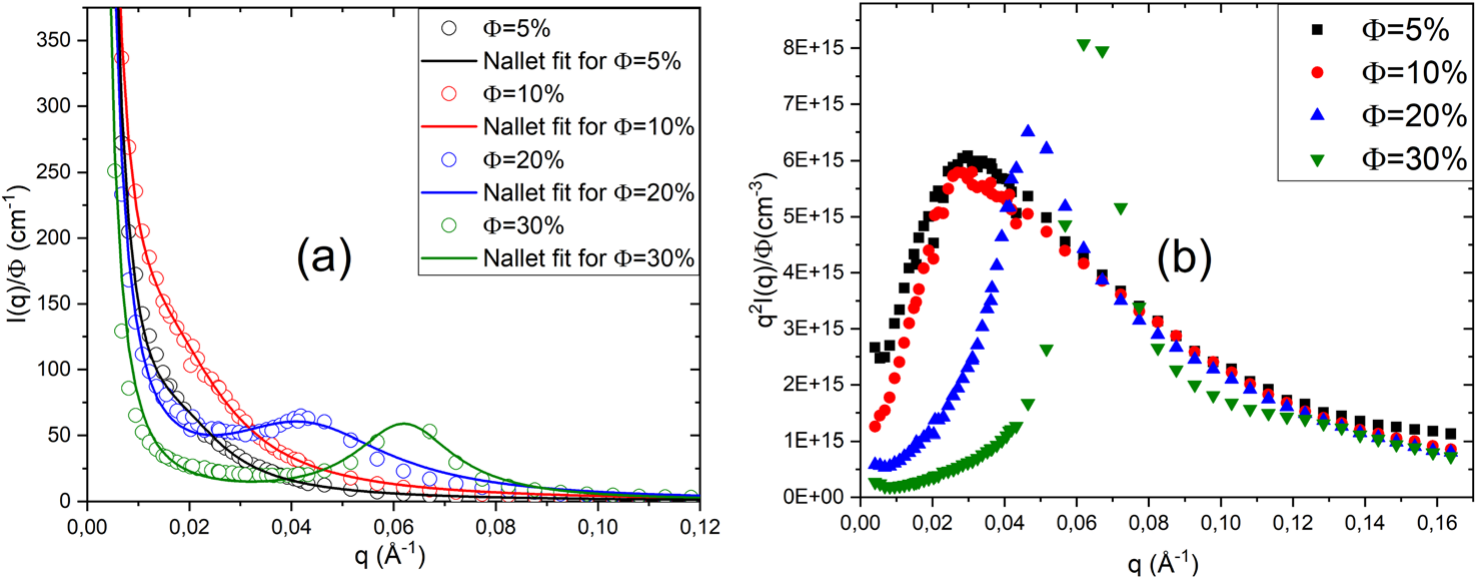
Scattered intensity *I*(*q*)/phi of neutral lamellar phases at different volume fractions *ϕ* (a), fitted with Nallet's theory, and representation *q*^2^*I*(*q*)/*ϕ* (b).

Fig. 2 presents the scattered intensity *I*(*q*) obtained from small-angle neutron scattering (SANS) for a system composed of a ternary mixture of two non-ionic surfactants, TX_100_ and TX_35_, and water, prepared in the lamellar phase. The mass ratio TX_35_/TX_100_ = 1.26 is fixed for all samples, while the volume fraction *ϕ* is varied from 5% to 30%. All the scattered intensity curves obtained from the experiment are fitted using Nallet's theory (eqn (5)). This theoretical model accounts for small-angle neutron scattering in lamellar phases by considering both the local disorder between membranes and their regular stacking. It describes the scattered intensity *I*(*q*) using two contributions: one related to local membrane fluctuations and another corresponding to the Bragg peak, which indicates lamellar order.

According to Fig. 2(a), at low volume fractions (*ϕ* = 5% and *ϕ* = 10%), the scattered intensity decreases monotonically with the scattering vector *q*, with no Bragg peak observed, indicating a disordered state lacking clear lamellar organisation. In contrast, at *ϕ* = 20%, a distinct Bragg peak appears in the scattering curves, suggesting the formation of periodic lamellar structures. This peak shifts towards higher *q* values, *i.e.*, towards smaller distances (*q* = 2π/*d*), and becomes more intense at *ϕ* = 30%, implying that lamellar correlations increase and the lamellae become closer to one another as the volume fraction rises.

To verify that the characteristics of the bilayers remain unchanged as the volume fraction varies, we present in Fig. 2(b) the appropriate representation for bilayers, *q*^2^*I*(*q*)/*ϕ* as a function of *q* at different volume fractions *ϕ*. According to this figure, at low *q* values, the spectra do not overlap, which is due to interactions within the system becoming more significant as the volume fraction *ϕ* increases. However, at high scattering vectors *q*, the spectra overlap, indicating that the bilayer morphology remains unchanged despite variations in the volume fraction *ϕ*.

In Table 2, we present the parameters obtained from fitting the scattered intensity curves *I*(*q*) (Fig. 2(a)) using the Nallet model at different volume fractions *ϕ*. According to Table 2, at a low volume fraction (*ϕ* = 5%), the parameter *A*_*z*_, associated with local concentration fluctuations within the membrane plane, is high, as is the lateral correlation length *ξ*_p_. This indicates that the system is dominated by disordered fluctuations, with no stable lamellar structure. As the volume fraction increases from 5% to 30%, both *A*_*z*_ and *ξ*_p_ decrease, suggesting that local concentration fluctuations within the membrane plane become progressively less significant, reflecting a reduction in small-scale disorder. Conversely, *A*_B_ and *ξ*_*l*_ are very small at *ϕ* = 5%, but increase progressively with higher volume fractions, indicating that the lamellar layers become more organised and more strongly correlated. The appearance and subsequent enhancement of the Bragg peak confirm the transition towards a structured lamellar phase, where the membranes are regularly spaced at increasingly well-defined distances. Thus, the evolution of these parameters highlights the transition from a disordered state at low *ϕ* to a stable and well-organised lamellar structure at *ϕ* = 30%.

**Table 2:** Fitted parameters from Nallet's model at different volume fractions *ϕ*

*ϕ*	*A*_*z*_ (cm^−1^)	*ξ*_p_ (Å)	*A*_B_ (cm^−1^)	*ξ*_*l*_ (Å)	*q*_0_
5%	14 531.6 ± 151.20	1033.03 ± 206.2	11.31 ± 1.18	2.43 × 10^−7^ ± N.D.	0.0091 ± 0.0001
10%	2114 ± 157.13	337.43 ± 17.54	72.32 ± 3.20	79.48 ± 6.45	0.1187 ± 0.0001
20%	760.77 ± 19.02	245.61 ± 16.52	50.51 ± 2.75	98.01 ± 3.08	0.04087
30%	493.14 ± 20.26	181.59 ± 7.40	62.55 ± 3.12	109.53 ± 5.68	0.06207

In order to confirm that the structure of our samples corresponds to a lamellar phase and that this structure remains unchanged with variations in the volume fraction *ϕ*, we consider the key parameters of this phase: the interlamellar distance *d* and the bilayer thickness *δ*. To determine the bilayer thickness *δ*, we base our analysis on a diluted case in which the interaction between membranes is very weak. In such a case, the scattered intensity for a homogeneous membrane of thickness *δ* can be expressed as:^27^10
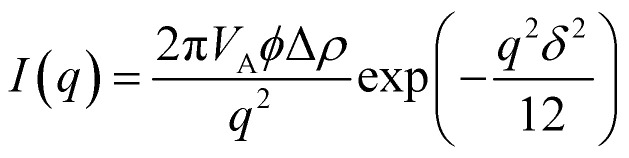
where *q* is the scattering vector, *ϕ* is the volume fraction, *V*_A_ is the membrane volume (*V*_A_ = 3 × 10^−7^ cm^3^), and Δ*ρ* is the electron density contrast (or neutron scattering length density contrast) between the membrane and the solvent (Δ*ρ* = 6 × 10^10^ cm^−2^).

This equation represents the form-factor contribution of a single bilayer and is particularly relevant at higher *q* values, allowing a direct estimation of *δ*. In our system, although the main scattering features are dominated by interlamellar correlations, this form-factor contribution provides a precise measure of the bilayer thickness in the weak-interaction regime.^50,57,58^ In an ideal lamellar system consisting of bilayers separated by solvent layers, the interlamellar distance *d* is related to the volume fraction *ϕ*^33,59,60^ by:11
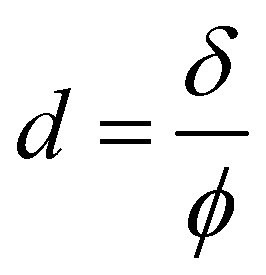


**Fig. 3:**
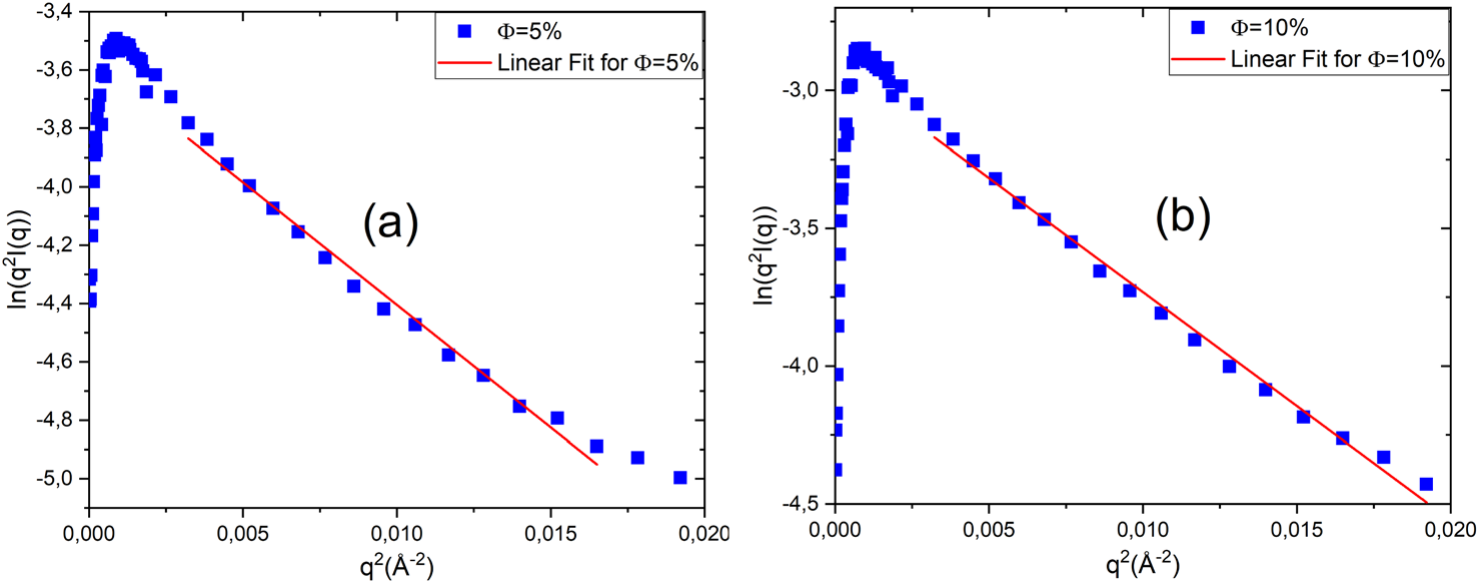
Plot of ln(*q*^2^*I*(*q*)) *versus q*^2^ at two volume fractions: *ϕ* = 5% (a) and *ϕ* = 10% (b).

**Fig. 4:**
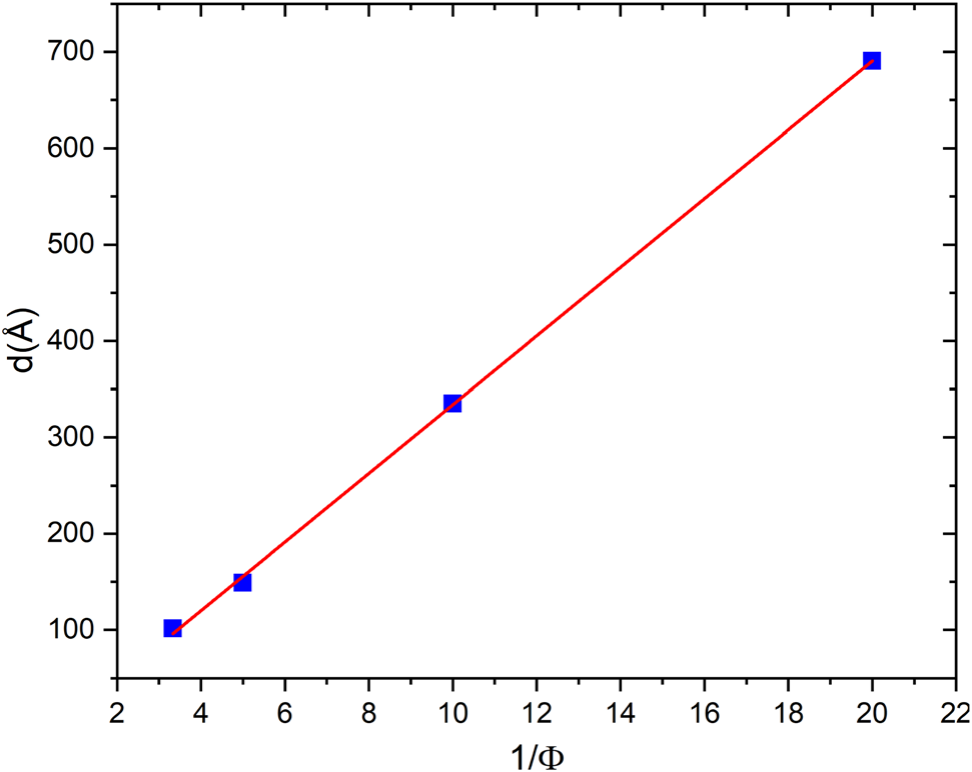
Dependence of interlamellar distance *d* on the inverse volume fraction 1/*ϕ* for a neutral lamellar phase.

To estimate the bilayer thickness, Fig. 3 shows the plot of ln(*q*^2^*I*(*q*)) *versus q*^2^ for low volume fractions normalising by *ϕ* to removes concentration dependence(*ϕ* = 5% and *ϕ* = 10%). Fitting these data with eqn (10) yields a bilayer thickness of approximately *δ* ≈ 32 Å. To clarify, the linear fits in this figure were performed over specific *q*-ranges corresponding to the linear regime of the experimental data. For *ϕ* = 5%, the fitting was carried out in the region 0.063 ± 0.05 Å^−1^ ≤ *q* ≤ 0.138 ± 0.05 Å^−1^, and for *ϕ* = 10%, in the region 0.055 ± 0.03 Å^−1^ ≤ *q* ≤ 0.141 ± 0.05 Å^−1^. The uncertainties reflect the variability observed from repeated fittings over neighbouring *q*-intervals, confirming the robustness and reproducibility of the analysis. Regarding the interlamellar distance *d*, it is determined from the position of the first correlation (Bragg) peak, *q*_max_, in the scattered intensity profile *I*(*q*). For high volume fractions (*ϕ* = 20% and *ϕ* = 30%), the interlamellar spacing is obtained using the Bragg condition for a one-dimensional periodic lamellar structure, expressed as 
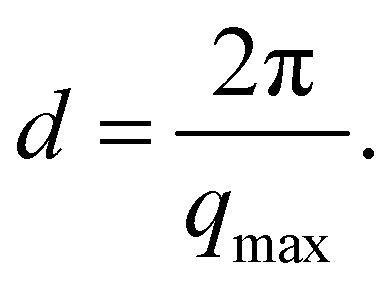
 This relation is widely used in the analysis of lamellar mesophases and is supported by previous studies.^61^ However, for low volume fractions (*ϕ* = 5% and *ϕ* = 10%), the Bragg peak does not yet appear in the scattered intensity curves. To determine the interlamellar distance *d* under these conditions, we use the classical geometric law for lamellar phases (eqn (11)). To clarify, in applying the classical geometric relation for low volume fractions (*ϕ* = 5% and *ϕ* = 10%), an ideal lamellar system is assumed, composed of parallel, planar bilayers of uniform thickness *δ*. This approximation is valid in the dilute limit, where inter-lamellar interactions are weak. Furthermore, the interlamellar distances obtained using this approach are consistent with those calculated from *q*_0_ using Nallet's equation, confirming that the values reported accurately represent the spacing between bilayers in our system. Fig. 4 presents *d* as a function of the inverse volume fraction 1/*ϕ*. The interlamellar distance increases linearly with 1/*ϕ*, in agreement with the expected behaviour for an ideal lamellar swelling, where the stacking of bilayers is governed purely by geometric constraints. From the slope of this linear dependence, the bilayer thickness is estimated to be *δ* ≈ 35 Å, which is consistent with values reported for other lamellar phases of non-ionic surfactants.^26^ Moreover, the increase in *d* with 1/*ϕ* indicates that the spacing between membranes decreases as the volume fraction *ϕ* increases, which is expected since the number of membranes within the system increases with *ϕ*.

In Table 3, we present the Caille parameter *η* and the compression modulus *B̄* (Pa) at different volume fractions *ϕ*. These two parameters were calculated using eqn (3) and (4). The Caille parameter *η* is a dimensionless quantity that measures the intensity of thermal fluctuations of bilayers in a lamellar phase. We observe that *η* decreases as the volume fraction *ϕ* increases. This is due to a reduction in the thermal fluctuations of the bilayers and an increase in the rigidity of the lamellar stacking. Conversely, the compression modulus *B̄* increases with increasing *ϕ*. This modulus measures the rigidity of the lamellar stacking against compression perpendicular to the bilayers. As the volume fraction increases, the bilayers become closer to each other, which strengthens the interlamellar repulsive interactions (in the case of a neutral lamellar phase, these are represented by the Helfrich interactions). This increase in interactions reduces the fluctuations and makes the lamellar stacking more rigid, resulting in an increase in the compression modulus *B̄* and a decrease in the Caille parameter *η*.

**Table 3:** Caille parameter and compression modulus as a function of volume fraction *ϕ*

	*η*	*B̄* (Pa)
*ϕ* = 5%	1.2	7.14 × 10^3^
*ϕ* = 10%	1.08	1.13 × 10^4^
*ϕ* = 20%	0.85	8.25 × 10^4^
*ϕ* = 30%	0.65	2.63 × 10^5^

### 4.3. Effect of charge on the structural properties of a lamellar phase

In this section, we investigate the effect of charge on the structural properties of the lamellar phase at different volume fractions *ϕ*. For this purpose, we compare the scattered intensities *I*(*q*) of a neutral system (TX_100_, TX_35_, and water) with those of the same system rendered charged by the addition of a fixed amount of CpCl (*Γ* = 1%).

**Fig. 5:**
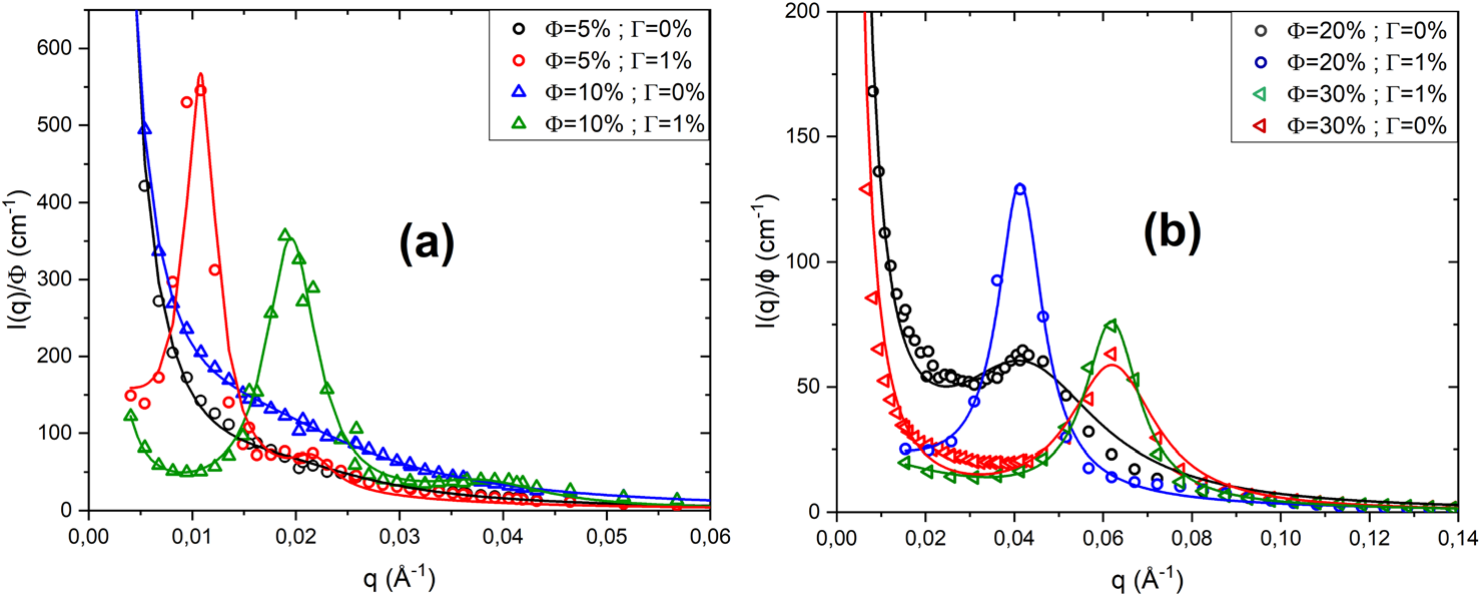
Scattered intensity *I*(*q*)/*ϕ* from SANS for neutral and charged lamellar systems at low (a) and high (b) volume fractions *ϕ*, fitted with Nallet's theory.

Fig. 5 shows the scattered intensity *I*(*q*)/*ϕ* obtained from small-angle neutron scattering for a neutral lamellar system and a lamellar system with a fixed charge (*Γ* = 1%), for low volume fractions (Fig. 5(a)) and high volume fractions (Fig. 5(b)). All the scattered intensity curves are fitted using Nallet's theory (eqn (5)). As shown in Fig. 5(a), for a neutral lamellar phase at low volume fraction *ϕ*, the scattered intensity *I*(*q*) decreases monotonically with *q*, with no Bragg peak formation. In this case, the membranes are weakly correlated and exhibit strong thermal fluctuations, leading to a disordered stacking. When a charge of *Γ* = 1% is introduced, the bilayers become highly correlated because the CpCl ions adsorbed on the bilayer surfaces introduce long-range electrostatic repulsive interactions, which add to the Helfrich interactions. This enhanced repulsion reduces thermal fluctuations and stabilises the interlamellar spacing, resulting in a more regular membrane stacking.

For a concentrated neutral lamellar system (*ϕ* = 20% and *ϕ* = 30%), the Bragg peak is already present in the scattered intensity curves, indicating a regular membrane stacking. When a charge is introduced by adding a fixed amount of CpCl (*Γ* = 1%), the Bragg peak becomes more intense, suggesting enhanced correlations between the bilayers due to the additional electrostatic repulsion induced by the charged surfactants. This increase in peak intensity reflects a more pronounced lamellar order, with the membranes adopting a more regular and well-defined stacking. For *ϕ* = 20%, the Bragg peak shifts to lower *q* values, indicating an increase in the interlamellar spacing in the charged system. In contrast, for *ϕ* = 30%, where the membranes are already very close to each other, the addition of charge (*Γ* = 1%) hardly changes the position of the Bragg peak, indicating that the interlamellar spacing remains almost unchanged between the neutral and charged systems.

In Table 4, we present the parameters obtained from fitting the scattered intensity curves *I*(*q*) using Nallet's model for both neutral and charged lamellar phases at different volume fractions *ϕ*.

**Table 4:** Parameters obtained for neutral and charged systems at different volume fractions *ϕ*

*ϕ*	*A*_*z*_ (cm^−1^)	*ξ*_p_ (Å)	*A*_B_ (cm^−1^)	*ξ*_*l*_ (Å)	*q*_0_
5%	102.15 ± 9.38	55.37 ± 4.26	546.33 ± 10.85	628.84 ± 25.98	0.0105
10%	44.05 ± 10.13	23.50 ± 7.24	326.93 ± 17.27	381.38 ± 35.15	0.0189
20%	21.76 ± 4.27	29.99 ± 7.94	122.82 ± 3.39	162.48 ± 7.84	0.0413
30%	22.58 ± 3.73	35.27 ± 7.33	72.31 ± 1.74	133.66 ± 5.77	0.0612

**Fig. 6:**
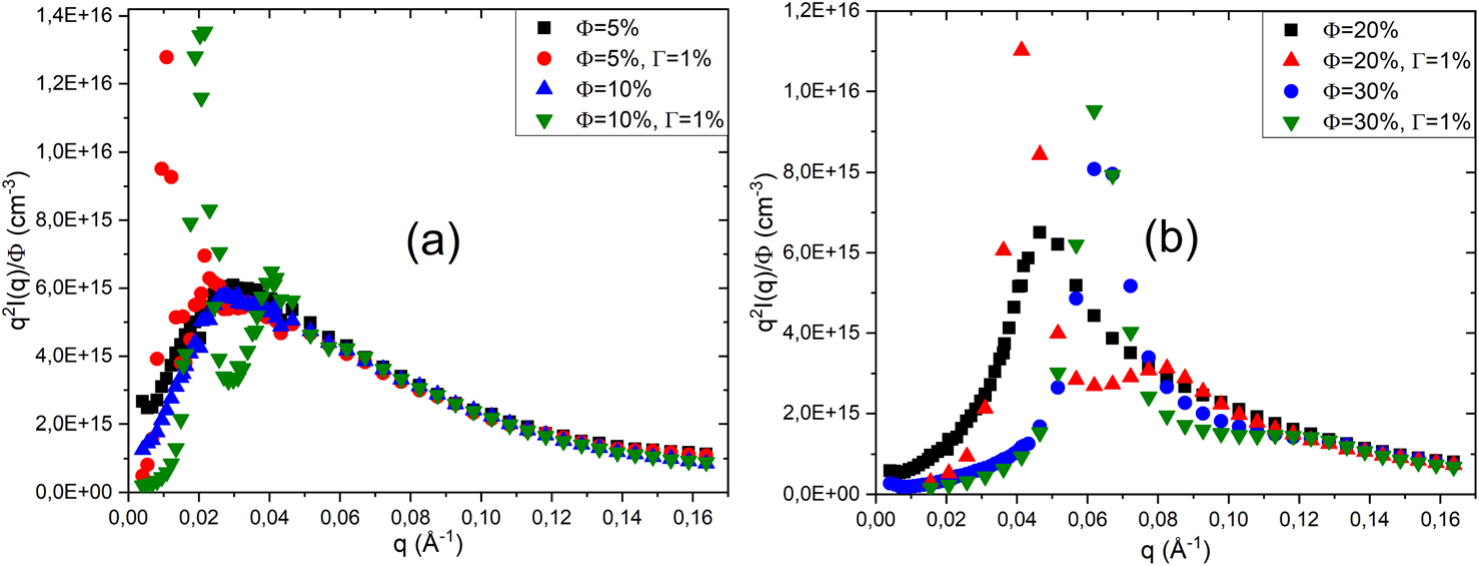
Representation of *q*^2^*I*(*q*)*ϕ versus q* for neutral and charged lamellar phases at various volume fractions: diluted (a) and concentrated (b) cases.

According to this table, for all volume fractions *ϕ*, the two parameters *A*_*z*_ and *ξ*_p_ decrease when a small amount of charge is added (*Γ* = 1%). This indicates that thermal fluctuations within the membrane plane become significantly reduced. Conversely, the addition of charges increases the parameters *A*_B_ and *ξ*_*l*_, suggesting that interlamellar correlations are strengthened and the lamellar order becomes more pronounced. These observations also demonstrate that the electrostatic interactions introduced by CpCl surfactants adsorbed on the membrane surfaces enhance correlations, stabilise the stacking, and reduce the effect of thermal fluctuations, thereby making the lamellar stacking more rigid and better organised. To verify that the morphology of the lamellar phase does not change upon the addition of charges, Fig. 6 shows the representation of the scattered intensity *q*^2^*I*(*q*)*ϕ* as a function of *q* for both neutral and charged lamellar phases at different volume fractions *ϕ*.

As observed, whether in the diluted case (Fig. 6(a)) or the concentrated case (Fig. 6(b)), at a given volume fraction *ϕ*, the curves do not overlap at low *q* values. This is due to the fact that the interactions within the system differ between the neutral and charged lamellar phases, the interactions in the charged lamellar phase being much stronger than in the neutral one. Conversely, irrespective of the volume fraction *ϕ*, the curves overlap at high *q* values. This indicates that the samples retain the same lamellar morphology, even after adding a small amount of charge (*Γ* = 1%). At higher concentrations (*ϕ* = 10% and *ϕ* = 20%), a second Bragg peak becomes clearly visible in the scattering intensity profiles of the charged systems, indicating the presence of higher-order reflections (Fig. 5). The multiplication by *q*^2^ in the Porod representation (*q*^2^*I*(*q*) *versus q*) amplifies these reflections, making the second peak more distinct. The appearance of this second Bragg peak, located at a *q*-position corresponding to an integer multiple of the first-order reflection, confirms the development of a long-range ordered lamellar structure. This behaviour suggests that charge addition enhances the electrostatic interactions between the surfactant headgroups, promoting a more regular stacking of the lamellae and improving interlayer correlation.

**Fig. 7:**
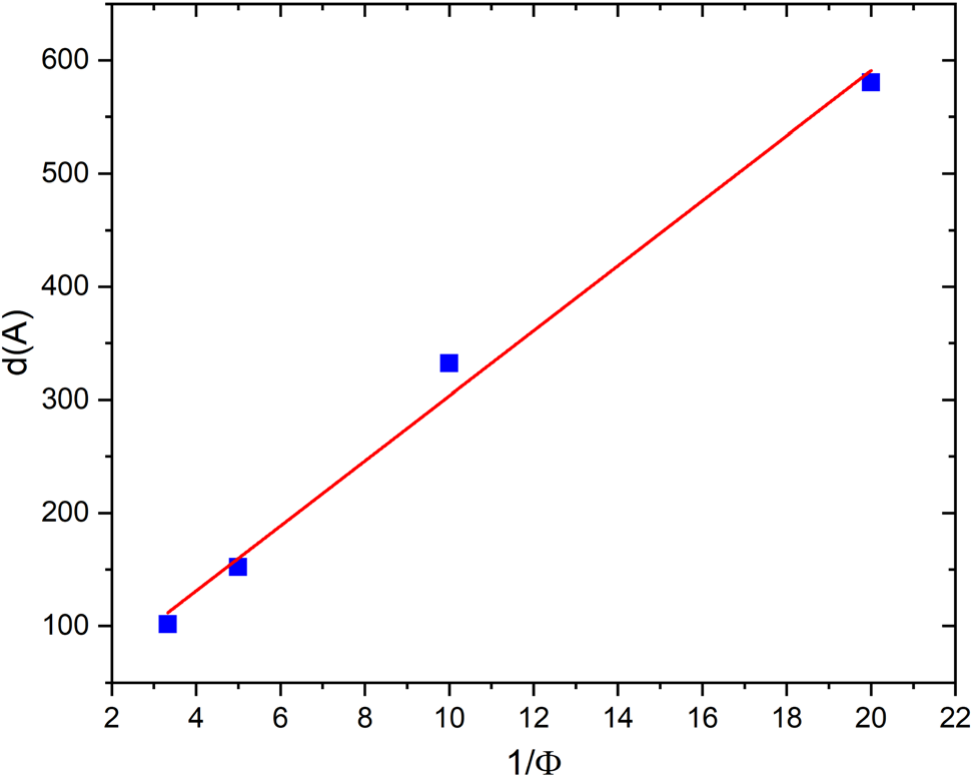
Dependence of interlamellar distance *d* on the inverse volume fraction 1/*ϕ* for a charged lamellar phase.

In Fig. 7, we present the variation of the interlamellar distance *d* as a function of the inverse volume fraction 1/*ϕ* for a charged lamellar phase. The interlamellar distances *d* are determined from the position of the Bragg peak shown in Fig. 5. From this figure, we observe a linear dependence of the interlamellar distance *d* on the inverse volume fraction 1/*ϕ*, which also confirms the presence of a lamellar phase in this system, even in the presence of charges. The structure corresponds to a lamellar arrangement undergoing simple swelling upon water addition. The straight line that best fits the experimental data points (according to the equation *d* = *δ*/*ϕ*) yields a dry thickness of *δ* = 30 ± 1 Å, a value almost identical to that obtained by the same method for the neutral bilayer.

One last aspect deserves attention. Our findings are in good agreement with previous investigations on weakly charged bilayers. Zou and Hoffmann demonstrated that introducing a small amount of ionic surfactant (SDS) into a lamellar phase composed of LA 070-EHG reduced the amplitude of bilayer undulations, confirming that neutral lamellar phases are primarily stabilized by Helfrich repulsion.^62^ Similarly, Schomäcker *et al.* reported that the addition of SDS to a nonionic C_12_E_4_/water/decane lamellar system slightly decreased the Bragg spacing, as a consequence of the suppression of membrane undulations by electrostatic interactions. These interactions effectively “freeze” the fluctuating bilayers and enhance interlamellar correlations.^63^ Comparable behaviour was also observed by von Berlepsch *et al.* who demonstrated that adding small amounts of ionic surfactant to a nonionic lamellar phase (C_10_E_3_/SDS) induces a transition from undulation-stabilized to electrostatically stabilized lamellae.^64^

### 4.4. Effect of polymer (PEO-2m) on the structural properties of a lamellar phase

In this section, we investigate the effect of adding a telechelic polymer with two terminal groups, PEO-2m, at different volume fractions *ϕ*. For this purpose, we compare the scattered intensities *I*(*q*) of a neutral system (TX_100_, TX_35_, and water) with those of the same system after the addition of a fixed amount of PEO-2m polymer (*Ψ* = 10%).

**Fig. 8:**
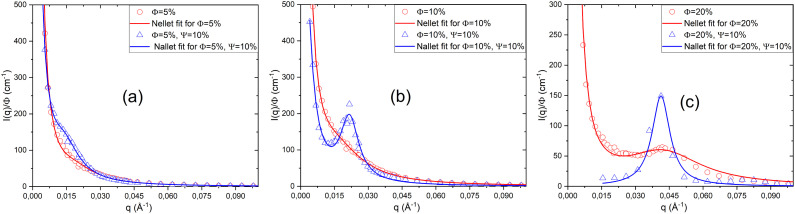
Scattered intensity *I*(*q*)/*ϕ* from SANS for neutral and PEO-2m containing lamellar systems at various volume fractions *ϕ*, fitted with Nallet's theory.

Fig. 8 shows the scattered intensity *I*(*q*)/*ϕ* obtained from small-angle neutron scattering for both the neutral lamellar system and the lamellar system containing PEO-2m (*Ψ* = 10%), at three volume fractions: *ϕ* = 5%, *ϕ* = 10%, and *ϕ* = 20%. All scattering curves were fitted using Nallet's theory (eqn (5)).

For low volume fractions (*ϕ* = 5% and *ϕ* = 10%, Fig. 8(a) and (b)), the scattered intensity *I*(*q*)/*ϕ* of the neutral system decreases monotonically, indicating a disordered state with no well-defined lamellar organisation. At *ϕ* = 5%, the scattered intensity remains monotonic even after the addition of PEO-2m, showing that the bilayers remain disordered. In contrast, at *ϕ* = 10%, the addition of PEO-2m (*Ψ* = 10%) induces the appearance of a Bragg peak, indicating the formation of periodic lamellar structures. For the higher volume fraction *ϕ* = 20% (Fig. 8(c)), where a Bragg peak is already present in the neutral system, the addition of the polymer enhances this peak, which becomes more intense and narrower. This suggests a reinforcement of lamellar order and stronger inter-bilayer correlations. At the microscopic scale, PEO-2m can decorate a bilayer when the interlamellar spacing is larger than its radius of gyration, introducing steric repulsive interactions. Conversely, when this distance is smaller or comparable to the radius of gyration, the polymer can bridge two bilayers, generating attractive interactions. These combined attractive and repulsive contributions strengthen interlamellar correlations, reduce thermal fluctuations, and stabilise the interlamellar spacing, leading to a more regular and better-organised lamellar stacking. In a highly diluted system (*ϕ* = 5%), however, strong thermal fluctuations and the lack of long-range order prevent effective polymer bridging, making lamellar phase stabilisation impossible. In Table 5, we present the parameters obtained from fitting the scattered intensity curves *I*(*q*) (Fig. 5) using Nallet's model for a neutral lamellar phase and a lamellar phase containing the PEO-2m polymer, at different volume fractions *ϕ*.

**Table 5:** Fitted parameters from Nallet's model for a neutral lamellar phase and a lamellar phase with PEO-2m polymer at different volume fractions *Φ*

*ϕ*	*A*_*z*_ (cm^−1^)	*ξ*_p_ (Å)	*A*_B_ (cm^−1^)	*ξ*_*l*_ (Å)	*q*_0_
5%	722.82 ± 44.21	161.06 ± 10.01	3.44 ± 3.21	3.92 × 10^−9^ ± N.D.	0.01378 ± 0.0001
10%	1123 ± 110.13	305.50 ± 21.70	172.63 ± 4.51	201.04 ± 8.76	0.02165
20%	21.76 ± 6.76	29.99 ± 8.18	144.75 ± 7.04	226.81 ± 8.64	0.04132

For a low volume fraction (*ϕ* = 5%), the parameters *A*_*z*_ and *ξ*_p_ decrease significantly after the addition of the polymer, indicating a notable reduction in thermal fluctuations within the plane of the membranes. However, the very low values of *A*_B_ and *ξ*_*l*_, even after the addition of the polymer, show that interlamellar correlations remain weak and that the lamellar order is not stabilised, which is consistent with the absence of a Bragg peak observed in Fig. 8(a). At *ϕ* = 10%, the effect of the polymer becomes more pronounced: *A*_B_ and *ξ*_*l*_ increase, reflecting strengthened interlamellar correlations and improved bilayer organisation, in agreement with the appearance of the Bragg peak in Fig. 8(b). Meanwhile, *A*_*z*_ and *ξ*_p_ decrease, suggesting that thermal fluctuations within the plane of the membranes are reduced, thereby promoting the establishment of a regular lamellar stacking. Finally, for a high volume fraction (*ϕ* = 20%), the effect of the polymer is even more marked: the strong decrease in *A*_*z*_ and *ξ*_p_ confirms a significant suppression of thermal fluctuations, whereas the substantial increase in *A*_B_ and *ξ*_*l*_ indicates a much stronger lamellar order and an enhanced stabilisation of the interlamellar spacing. These results are consistent with Fig. 8(c), where the Bragg peak becomes more intense in the presence of the polymer.

**Fig. 9:**
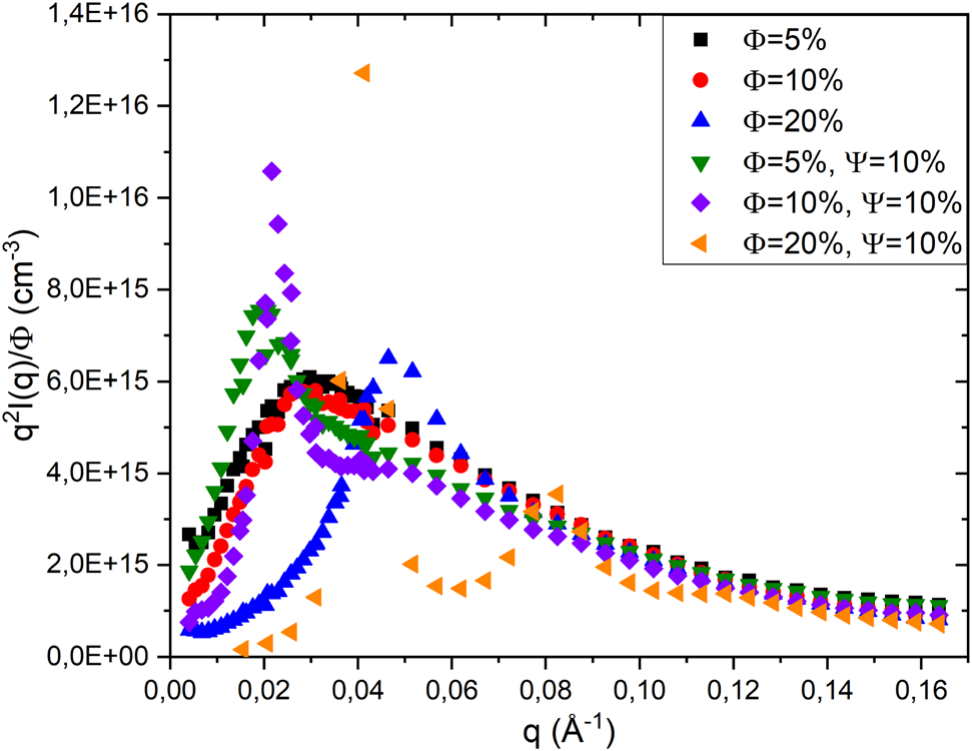
Representation of *q*^2^*I*(*q*)/*ϕ versus q* for neutral and PEO-2m containing lamellar phases at various volume fractions *ϕ*.

To confirm that the addition of a PEO-2m telechelic polymer (*Ψ* = 10%) does not alter the morphology of the lamellar phase but only affects the interactions within the system, Fig. 9 shows the representation of the scattered intensity *q*^2^*I*(*q*)/*ϕ* as a function of *q* for a neutral lamellar phase and a lamellar phase containing the PEO-2m polymer, at different volume fractions *ϕ*.

According to this figure, for the three volume fractions (*ϕ* = 5%, *ϕ* = 10%, and *ϕ* = 20%), the scattered intensities of the neutral and polymer-containing systems do not overlap at low *q* values, indicating that the polymer modifies the interactions between the bilayers. In contrast, at high *q* values, the intensity curves overlap even after the addition of PEO-2m (*Ψ* = 10%), demonstrating that the polymer does not change the morphology of the lamellar phase and that the bilayer structure is preserved. At *ϕ* = 20%, however, the presence of the telechelic polymer leads to the appearance of a second Bragg peak in the scattering intensity profile, reflecting the formation of higher-order reflections (Fig. 8). When the data are represented in the Porod form (*q*^2^*I*(*q*) *versus q*), this secondary peak becomes more distinct due to the amplification of higher-order contributions. The emergence of this second Bragg reflection at a *q*-position corresponding to a multiple of the first-order peak confirms the establishment of a more ordered lamellar morphology. The polymer chains, anchored to the surfactant layers through their hydrophobic ends, act as spacers and bridges between adjacent lamellae, thereby enhancing interlayer correlations and stabilising the long-range ordered lamellar structure.

## 5. Conclusion

In this study, small-angle neutron scattering experiments were carried out to investigate the effects of the volume fraction *ϕ*, the addition of charges through a cationic surfactant (CpCl), and the incorporation of a telechelic polymer with two end groups (PEO-2m) on the structure and stability of a lamellar phase composed of TX_100_ surfactant, TX_35_ co-surfactant, and water. All scattered intensity curves, *I*(*q*), were successfully fitted using the Nallet model, confirming the lamellar nature of the prepared samples. The analysis of these curves shows that, at low volume fractions, thermal fluctuations are significant, and the membranes are in a disordered state, lacking clear lamellar organisation. Increasing *ϕ* enhances interlamellar correlations and reduces thermal fluctuations, leading to a more rigid and better-organised lamellar stacking. The addition of a small amount of charge strengthens the repulsive interactions between the membranes by introducing electrostatic interactions, enabling the formation of an ordered lamellar phase even at low volume fractions *ϕ*. Regarding the addition of PEO-2m, this polymer induces both repulsive interactions (decoration effect) and attractive interactions (bridging effect). At very low volume fractions (*ϕ* = 5%), the addition of the polymer does not result in an ordered lamellar phase. However, at *ϕ* = 10%, it promotes the formation of an ordered lamellar structure, and at *ϕ* = 20%, where the phase is already ordered, the polymer further enhances the lamellar order.

Lamellar phases possess significant biological and technological relevance for instance, in membrane formation and in drug delivery systems, where they serve as carriers due to their bilayer structure and permeability properties. In dermal drug delivery,^65^ lamellar phases exhibit structural similarity to intercellular lipids, enabling them to solubilize large amounts of hydrophilic, hydrophobic, or amphiphilic drugs,^66,67^ enhance their stability,^68^ and improve targeting while reducing side effects.^69^ They also play an important role in the encapsulation and controlled release of biomacromolecules such as proteins and DNA.^70^ Therefore, understanding and controlling lamellar phase stability and interactions is crucial for optimising their performance in biomedical and pharmaceutical applications.

## Conflicts of interest

There are no conflicts to declare.

## Data Availability

The data generated and analyzed during this study are available from the corresponding author upon reasonable request.
